# Gen Z and HIV—Strategies for Optimizing the Care of the Next Generation of Adolescents Living with HIV

**DOI:** 10.3390/v15102023

**Published:** 2023-09-29

**Authors:** Inès Dufour, Yves Fougère, Tessa Goetghebuer, Marc Hainaut, Benoît Mbiya, Fatima Kakkar, Jean Cyr Yombi, Dimitri Van der Linden

**Affiliations:** 1 Department of Internal Medicine and Infectious Diseases, Cliniques Universitaires Saint-Luc, Avenue Hippocrate, 10, 1200 Brussels, Belgium; jean.yombi@saintluc.uclouvain.be; 2 Division of Infectious Diseases, Department of Pediatrics, CHU Sainte-Justine, Université de Montréal, Montréal, QC H3T 1J4, Canada; yves.fougere@umontreal.ca (Y.F.); fatima.kakkar@umontreal.ca (F.K.); 3 Centre d’Infectiologie Mère-Enfant (CIME), Department of Pediatrics, CHU Sainte-Justine, Université de Montréal, Montréal, QC H3T 1J4, Canada; 4 Department of Pediatrics, Centre Hospitalier Universitaire Saint-Pierre, Université Libre de Bruxelles, 1000 Brussels, Belgium; tessa_goetghebuer@stpierre-bru.be (T.G.); marc.hainaut@stpierre-bru.be (M.H.); 5 Pediatrics Department, Faculty of Medicine, University of Mbujimayi, Mbujimayi 06201, Democratic Republic of the Congo; bembiya@gmail.com; 6 Sickle Cell Reference Center, Clinique Pédiatrique de Mbujimayi, Pediatrics Clinic of Mbujimayi, Mbujimayi 06201, Democratic Republic of the Congo; 7 Institut de Recherche Expérimentale et Clinique, Université Catholique de Louvain, 1348 Brussels, Belgium; dimitri.vanderlinden@saintluc.uclouvain.be; 8 Pediatric Infectious Diseases, Service of Specialized Pediatrics, Department of Pediatrics, Cliniques Universitaires Saint-Luc, Université Catholique de Louvain, 1200 Brussels, Belgium

**Keywords:** HIV, adolescents, care, treatment

## Abstract

The management of adolescents living with HIV represents a particular challenge in the global response to HIV. The challenges specific to this age group include difficulties engaging and maintaining them in care, challenges with transition to adult care, and limited therapeutic options for treatment-experienced patients, all of which have been jeopardized by the COVID-19 pandemic. This paper summarizes some of the challenges in managing adolescents living with HIV, as well as some of the most recent and innovative therapeutic approaches in this population.

## 1. Introduction

Generation Z comprises people born between the mid-1990s and 2010 (the delimitation may vary between authors) and who are the first generation that has always evolved with new technologies. The adolescents living with HIV (ALHIV) that we are currently following belong to this generation. Their realities and needs may differ from those of previous generations, and care must be tailored accordingly [1]. Due to early detection and treatment of the HIV infection, improved access to combination antiretroviral therapy (cART), and more effective treatment regimens, the number of ALHIV globally has increased, reaching approximately 1.75 million worldwide [2]. The first generation of vertically infected adolescents to be transferred to HIV adult care providers had complex medical treatment histories [3]. They were the youngest and most experienced patients treated by adult physicians. Multiple resistant mutations were selected in these patients because of sequential therapies with single or old dual drug regimens due to the limited treatment options available at that time. ALHIV who are currently transitioning to adult care are different, as the majority were initiated with potent cART [4,5]. However, despite effective therapy, ALHIV remain particularly at risk of poorer health outcomes compared to adults, with lower rates of retention in care, lower adherence, suboptimal virological control [6,7,8] and higher overall mortality [9,10]. Given these challenges, unique strategies specific to the adolescent context are needed to improve long-term outcomes for this Z generation of ALHIV. In this paper, based on the current literature, international guidelines and our own personal experience, we review some of the challenges specific to ALHIV care, and some of the newer strategies to address them.

## 2. Heterogeneity of Adolescents Living with HIV

There is a wide spectrum in the timing of diagnosis and entry into care for ALHIV. We usually distinct two groups of patients: ALHIV with vertically acquired HIV (mother-to-child transmission) and those who acquired HIV horizontally (through sexual activities, transfusion, injecting drugs or sexual abuses). The first group acquired HIV during early childhood (in utero, at delivery or during breastfeeding) with longer exposition to the virus leading to higher disease burden, more complications, increased risk of mortality, and it was more likely to have prior experience with antiretroviral therapy. These patients may also have developed resistance to antiretroviral drugs, which can limit their options for future treatment regimens [11].

In 1989, a quarter (25%) of babies born to mothers living with HIV were infected with the virus, and by the age of 2 years, 25% of them had sadly passed away due to HIV-related complications [12]. In 1994, the Pediatric AIDS Clinical Trial Group Study reported a 67.5% reduction in the transmission of HIV from mother to child when pregnant women received prophylactic antenatal, intrapartum, and postnatal zidovudine [13]. Since then, numerous studies have provided strong evidence for refining these interventions, leading to annual transmission rates as low as 0% in many parts of the United States, and even the successful elimination of mother-to-child transmission of HIV in several countries worldwide. However, despite these achievements, the global elimination of vertical HIV transmission remains an ongoing challenge, primarily due to socioeconomic factors such as the high cost of antiretroviral medications [12].

## 3. Preferred Antiretrovirals for Adolescents Living with HIV

All first-line regimens currently include two Nucleoside Reverse Transcriptase Inhibitors (NRTIs) together with a drug from a different class (Non-Nucleoside Reverse Transcriptase Inhibitors (NNRTI), Protease Inhibitors (PI) or integrase inhibitors (INI)). Dolutegravir (DTG), a second-generation of INI plus 2 NRTI combination, is the preferred option [11,14,15,16]. Clinical trials, one of which the ODYSSEY Trial [17], have demonstrated that DTG-based ART was superior to PI-based regimens. Table 1 compares the different guidelines (PENTA/EACS, NIH, and WHO) concerning initial regimen in ALHIV.

Simple tablet regimen, with fixed-dose, once-daily combinations (cART) with high barriers to resistance are preferable, with minimal side effects (e.g., gastrointestinal) to promote adherence. 

Physiological changes like puberty or rapid growth that occur in adolescence result in altered pharmacokinetics. Therefore, while it is generally appropriate for post-pubertal adolescents to be dosed with cART according to adult guidelines, adolescents in early puberty should receive adapted dosages according to the pediatric guidelines with dosing based on their weight and Tanner stage [18]. 

HIV drug-resistance testing is recommended at entry into care for people with HIV to guide the selection of the initial cART regimen [11]. Genotyping is also recommended for resistance testing in adolescents who experience virologic failure or suboptimal virological control while on a first or second cART drug regimen [11]. 

In 2021, the results of the NADIA study were published, showing that dolutegravir-based regimen was proven virologically noninferior to darunavir-based regimen as second-line therapy in a randomized African trial [19]. The findings support World Health Organization (WHO) guidance to use the integrase inhibitor DTG in second-line regimens.

For ALHIV who developed resistance to two classes of HIV medications, using a combination of darunavir/cobistat/emtricitabine (FTC)/tenofovir alafenamide (TAF) along with dolutegravir (DTG) can provide a powerful treatment option. This combination has a strong ability to prevent the development of drug resistance, with only two pills once daily [11]. 

## 4. Medication Side Effects

Another major challenge in ALHIV is the need to minimize side effects of ART, which is particularly challenging when treatment is initiated early in life. To avoid the risk of lactic acidosis and mitochondrial toxicity due to some old medications like stavudine, most experts now recommend a cART including mitochondria-friendly NRTIs (e.g., abacavir, tenofovir and lamivudine/emtricitabine) or even a nucleoside-sparing regimen, especially in patients previously affected with an episode of symptomatic hyperlactatemia. While lipodystrophy-inducing drugs are no longer used, dyslipidemia and insulin resistance remain concerns and must be regularly assessed for prompt management and control. ALHIV may also be at risk of low bone mineral density (BMD) and long-term osteoporosis. While this is thought to be due to specific ARVs, notably tenofovir and a PI-based regimen, other factors may affect BMD, including ethnicity, gender, genetic factors, dietary intake of vitamin D and calcium, weight-bearing physical activity, and pubertal development [20]. In ALHIV on tenofovir, especially those with boosted PI, renal function must be monitored as it is in adults, in order to detect increasing levels of serum creatinine, proteinuria or hypophosphatemia that may be a sign of renal proximal tubular dysfunction. Long-term cardiovascular risks associated with both HIV infection (resulting from endothelial dysfunction driven by inflammation) and specific antiretroviral drugs (such as abacavir and boosted PI) must be considered in the choice of ART given a patient’s other risk factors such as tobacco exposure, hypercholesterolemia, physical inactivity or obesity. Close cardio-vascular follow-up is encouraged, as well as efforts to promote a healthy lifestyle, including proper diet, physical activity, and tobacco avoidance. Integrase inhibitors (INI) are generally well tolerated but may be associated with weight gain (dolutegravir (DTG) and bictegravir (BIC)) and should be monitored closely [21], especially in the obesogenic environment currently affecting teenagers worldwide [22]. Other drugs have also been implicated in weight gain, including rilpivirine and tenofovir alafenamide [23]. However, weight gain in ALHIV, like in adults, is multifactorial, and the mechanism behind this weight gain remains not completely understood, in fact [24,25].

## 5. Key Complications Affecting Adolescents Living with HIV

Persistent inflammation is increasingly being recognized as a significant consequence of HIV infection, even despite cART initiation [26], with modification of the cytokine network and metabolic changes also linked with antiretrovirals. HIV infection results in oxidative stress and abnormal production of reactive oxygen species (ROS), causing endothelial dysfunction, tissue damage, and chronic inflammation [27]. Furthermore, Heany et al. showed in a longitudinal cohort of ALHIV an accelerated epigenetic ageing [28]. For the study, blood DNA methylation data from 60 adolescents with perinatally acquired HIV was analyzed and matched with 36 age-matched controls enrolled in the Cape Town Adolescent Antiretroviral Cohort Study (CTAAC). Using epigenetic clock software, the authors found that HIV status was associated with epigenetic accelerated ageing and age acceleration difference. Specifically, the HIV-positive group was a mean of 2.3 years older in epigenetic terms than controls, even though their chronological ages were well matched [28].

**Figure 1:**
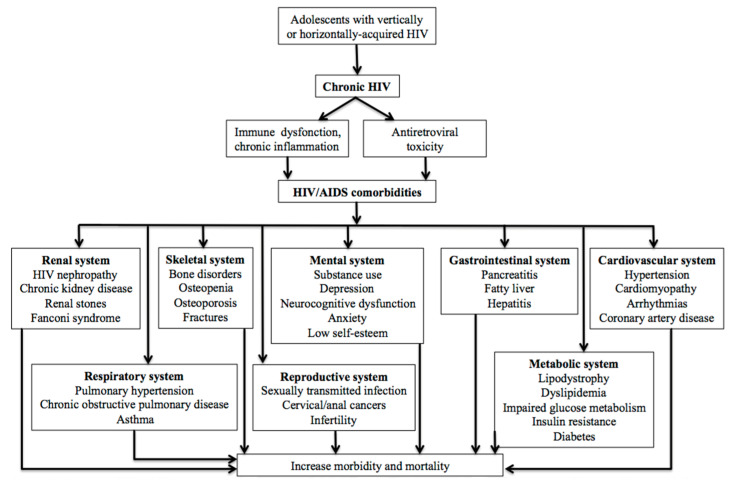
Complications affecting adolescents living with HIV.

Due to a combination of chronic inflammation and metabolic toxicity linked to cART, ALHIV are at risk of HIV-related comorbidities, including cardiovascular, liver, renal and bone diseases, diabetes, and mental health disorders (Figure 1). In a recent longitudinal study of ALHIV, 15% had impaired glucose tolerance and 33% had insulin resistance, both of which increased with exposure to cART [29]. 

Given the increasing evidence of cardiovascular involvement in HIV infection in adolescents, evaluation for possible cardiovascular complications should be emphasized in the care of ALHIV to allow early detection and effective management [30]. Cardiac abnormalities probably need to be screened regularly in ALHIV using a sensitive tool, such as strain echocardiography, to detect systolic dysfunction [31].

Elevated cholesterol levels are common in cART-treated HIV adolescents with greatest increases associated with use of boosted PI; however, there are no clear recommendations for management of cART-associated hyperlipidemia in adolescents. Management options include lifestyle and dietary changes. If lipid concentrations do not improve with these interventions, some studies have shown that treatment with statin appears to be safe and effective for ALHIV [32]. Many of the statins currently in use are metabolized by cytochrome P450 3A4 enzymes; therefore, the risk of drug interactions with PIs is significant. Based on pharmacokinetic and clinical data, atorvastatin and pravastatin are generally considered safe for HIV patients receiving cART. Rosuvastatin is generally safe if started at a low dose and titrated to a maximum of 20 mg per day. Fluvastatin, lovastatin, and simvastatin should be avoided in patients with HIV receiving cART [33]. More recently, the results of the REPRIEVE trial were published, showing that in HIV patients at low-to-moderate risk for cardiovascular disease who were receiving cART, those who received pitavastatin had a lower risk of a major adverse cardiovascular event than those who received placebo over a median follow-up of 5 years [34].

## 6. Neurodevelopmental Issues and Mental Health

Cognitive and executive functions may be impaired because of untreated chronic HIV infection, late onset of treatment, or adverse environments in early childhood [35,36]. Among teenagers who contract HIV at an early age, close to 60% meet criteria for a psychiatric disorder at some point in their lives [37].

Adolescents with poorly controlled HIV are at a higher risk of developing neuroAIDS-related issues. Consistent with its preferential effects on the fronto-striato-thalamo-cortical systems, HIV infection is marked by deficits in executive functions (e.g., planning), memory and psychomotor speed [38]. Furthermore, multiple family and contextual factors may also influence mental health. In many parts of the world, the majority of ALHIV are from ethnic minority families, living in impoverished communities affected by violence or substance abuse, all of which may influence mental health. Moreover, it is important to highlight the ongoing concern for some antiretrovirals and the risk of depression. Efavirenz (EFV) has been associated with depression in adults and especially in adolescents [39], and there are concerns about newer drugs (such as INI) due to their high level of penetration into the central nervous system and their potential neurocognitive side effects [40,41]. 

Adolescents living with HIV should undergo regular screening and monitoring for neuropsychological problems to ensure early detection and appropriate intervention. This can be achieved through a combination of clinical assessments, self-report measures, and standardized neuropsychological tests to identify specific areas of impairment in ALHIV. It is recommended to include comprehensive neuropsychological assessments that cover various cognitive domains such as attention, memory, executive functions, and psychomotor skills. These tests can provide valuable information about the extent and nature of neurocognitive deficits, aid in treatment planning, and monitor the effectiveness of interventions over time. Medication may be indicated for ALHIV who are experiencing significant neuropsychological problems that affect their daily functioning and quality of life, in parallel of tailored psychotherapy [42]. A multidisciplinary approach involving HIV specialists, neurologists, and mental health professionals is recommended to provide comprehensive care for ALHIV and address their neuropsychological needs [11].

## 7. Opportunistic Infections

Opportunistic infections refer to infections that occur more frequently or become more severe because of HIV-mediated immunosuppression. These infections were the first signs that alerted clinicians to the presence of AIDS. Examples of such infections include tuberculosis, toxoplasma encephalitis, Pneumocystis pneumonia, cytomegalovirus retinitis, and cryptococcal meningitis [43]. In general, the range of opportunistic infections occurring in ALHIV is more similar to what is observed in adults with HIV rather than in HIV-infected infants and young children [44,45]. For instance, cryptococcosis, which is less common among younger pediatric patients, becomes a more frequent infection among HIV-infected young adults in sub-Saharan Africa. Additionally, the incidence of tuberculosis increases during adolescence [46,47]. As teenagers have more social interactions, this wider social contact puts immunosuppressed young people at especially high risk of tuberculosis infection and disease [47,48]. 

Opportunistic infections, their treatment, and hospitalizations can have a significant impact on ALHIV schooling. Extended hospital stays, frequent medical appointments, and side effects of medications may result in missed school days, disrupted academic progress, and challenges in maintaining a consistent educational routine.

## 8. Sexual Development and Contraception Counseling

In a 5-year study of 983 HIV-infected children aged 6 to 18 years in the United States, it was found that HIV-infected children may experience delayed puberty and adrenarche compared with similarly aged HIV-uninfected children. Immunosuppression was associated with delayed pubertal onset in perinatally HIV-infected children [49].

Even though HIV infection alone does not preclude the use of any hormonal contraceptive method, medical conditions and medications used in ALHIV may influence contraceptive choices [50].

Long-acting reversible contraceptive (LARC) methods, such as intrauterine device (IUDs) and contraceptive implants, are ideal contraceptive methods for the adolescent population, because they are user-independent options with no need for regular adherence for effectiveness. Indeed, many HIV-infected adolescents are challenged by daily adherence to cART, so adding an additional medication to which they need to adhere may be undesirable [51]. 

Hormonal contraceptives are primarily metabolized in the liver by the cytochrome P450 system and it is known that antiretroviral agents have varying effects on this metabolic pathway. Special consideration is necessary for women who use some hormonal methods with some antiretroviral regimens (particularly those containing efavirenz and ritonavir-boosted PIs) [52].

In general, NRTIs do not appear to have significant interactions with hormonal contraceptives [50,51,52]. For NNRTIs, pharmacokinetic studies showed significant decreases in contraceptive hormone levels in women taking combined oral contraceptives (COCs) and efavirenz-contaning cART [53,54]. On the basis of primarily pharmacokinetic data, the efficacy of progestin injectables (DMPA) is likely not affected by NNRTIs. Furthermore, the efficacy of cART does not appear to be affected by the use of hormonal contraceptive methods on the basis of limited clinical data [55]. 

Table 2 summarizes the interactions of hormonal contraceptives with antiretroviral drugs. Up-to-date information regarding drug interactions with antiretroviral agents can be found within the regularly updated CDC, WHO, and NIH guidelines [11,56,57] or using software tools (e.g., HIV Drug Interactions, University of Liverpool, available at https://www.hiv-druginteractions.org/checker, accessed on 1 June 2023).

Hormonal contraceptive methods do not protect against sexually transmitted infections (STIs). Dual method use—the use of a condom in conjunction with a highly effective contraceptive method—should be a central component of reproductible health counseling for HIV-infected adolescents [58]. 

Some studies also showed that integration of family planning services within HIV care has resulted in an increase in contraceptive use [59]. Furthermore, reproductive health discussions need to be integrated with discussions on HIV care, because a reduction in HIV VL below the detection level is essential for a reduction in HIV transmission to partners and children. EACS proposes the U=U campaign (undetectable = untransmissible), which is potentially a motivational message to enhance adherence and prevent HIV transmission in sexually active adolescents [14].

It is important for ALHIV to disclose their HIV status to their sexual partners as it helps reduce the transmission of HIV [60]. However, the fear of stigma, violence, and rejection create barriers for these adolescents in disclosing their status. It is crucial to implement interventions that specifically address disclosure for ALHIV. These interventions should include education about consistent condom use, counseling on disclosure and negotiating safe sexual practices with partners, and understanding the significance of maintaining an undetectable VL to prevent HIV transmission. Providing youth-friendly services that are incorporated in HIV services is a promising approach to improve comprehensive sexuality education, equipping adolescents with the adequate knowledge to make informed decisions [61].

In our center in Belgium, we also propose post-disclosure support for the partner by the treatment team who can meet the couple and answer the partner’s questions to help the process and reduce fears.

## 9. Adherence Barriers

Like other chronic diseases, adherence remains the major driver of therapeutic success in patients living with HIV. However, adherence to treatment is particularly challenging for ALHIV. Adolescence is a time when youth want to comply and belong to their peer group and are particularly concerned about the stigma associated with HIV. Fear of being seen taking medication has been strongly associated with lack of adherence [62]. Adherence to treatment is directly affected by their activities (going out, sports, holidays). Adherence can be measured by self-report, affected by recall bias [63], or by VL measurement as a proxy. Rates of adherence vary from one study to another between 16% and 99% among teenagers globally [64]. A meta-analysis of ALHIV in 53 countries reported an adherence level of 84% in both Africa and Asia, based on either VL measurements or self-report [65]. In contrast, in a Quebec study on outcomes after transfer to adult care, the self-reported rate of excellent adherence among youth was only 40% [66]. Evidence-based practices in adults, such as community-based adherence support, use of mobile health (m-Health) platforms, nutrition support and group-adherence counseling need to be confirmed in adolescent populations [67]. 

## 10. Particularities of ALHIV Living in Low- and Middle-Income Countries

Adolescents facing challenges like lack of medical insurance, difficulties with work or school, family responsibilities, transportation issues to clinic visits, may struggle with adherence to their medical regimen. While these challenges can be found in well-resources countries, there are even more prevalent in resource-limited settings, especially those affected by social and political instability [68]. Moreover, there is a higher burden of additional health issues like tuberculosis, malaria, and malnutrition. The resulting polymedication and potential drug–drug interactions can further impact adherence. Lastly, the scarcity of healthcare professionals skilled in managing adolescent healthcare, including doctors, nurses, psychologists, social workers, and counselors, may further impact the adherence and the support needed for adolescents living with HIV in resource-limited settings [18]. 

## 11. Approaches to Optimize Care for ALHIV

Many innovations, such as long-acting agents, new delivery modalities (injectable and nanoparticles), and novel paradigms (immunotherapy or dual therapy), have been introduced to facilitate the administration of antiretroviral treatment to patients infected with HIV and improve their adherence and quality of life without altering the drug effectiveness.

### 11.1. Dual Therapy Regimens

We need to distinguish between two situations: (i) the treatment-naive patient and (ii) the virologically suppressed patient in whom we wish to simplify treatment. For the first situation, GEMINI 1 and 2 studies demonstrated non-inferiority at 96 weeks of dual therapy with DTG and lamivudine when compared to a cART of DTG and two NRTIs in treatment-naive HIV-1 infected adults [69]. Concerning the second situation, switching to a two-drug regimen is an option for some virologically suppressed people with HIV, with a large number of data for adults [70]. Reasons to consider switching include reduction in long-term toxicity, increasing tolerability and reduction in drug interactions. For example, the TANGO trial demonstrated that the dual therapy of DTG and lamivudine was noninferior in maintaining virologic suppression vs a TAF-based regimen at week 48, with no virologic failure or emergent resistance reported with DTG/lamiduvine, supporting it as a simplification strategy for virologically suppressed people with HIV-1 [71]. In the same line, the SALSA study showed that switching to DTG/lamivudine was noninferior to continuing cART for maintaining virologic suppression at week 48 with no observed resistance [72].

Recent pediatric evidence was presented on the dual therapy regimen. Smile Penta clinical RCT compared a once-daily INSTI (administered with darunavir/ritonavir) to standard of care among 318 HIV-1 infected virologically suppressed children aged 6 to 18 years and reported a non-inferiority at 48 weeks of a once-daily INSTI (Elvitegravir or DTG) administered with darunavir/ritonavir compared to standard of care, with a similar safety profile [73].

### 11.2. Intermittent Antiretroviral Therapy

Studies have investigated the use of intermittent treatment, especially weekends-off cART in HIV-suppressed patients. The BREATHER trial included 199 HIV-infected young patients (aged 8–24 years) with undetectable viral load for at least 12 months on efavirenz-based ART. The 99 patients in the test arm adopted a 5-day-on 2-day-off pattern: at 48 weeks, there was no significant difference between the two groups in terms of virological failure or resistance acquisition [74]. A more recent study called QUATUOR, including adult patients (median age of 49 years), found that a 4-day-on, 3-day-off pattern was non-inferior to the continuous pattern (7 days on) at 96 weeks [75]. Better-quality studies focusing on adolescents with long-term follow-up (96 weeks or more) are needed to determine the validity of intermittent treatment and the optimal regimens and monitoring to be used in the management of virologically suppressed ALHIV [76].

### 11.3. Long-Acting Antiretrovirals

A new and much anticipated strategy to mitigate the challenges associated with poor adherence, drug side effects, and drug fatigue is the use of long-acting injectable antiretrovirals [77]. Several RCTs conducted in HIV-infected adults demonstrated that the combination of intramuscular long-acting (IM-LA) cabotegravir (CAB) and rilpivirine (RPV) administered every 4 or 8 weeks is well tolerated and non-inferior to continuous oral therapy [78,79,80,81]. What emerges from these studies is that IM-LA seems to have a good level of efficacy, few side effects, and especially a high level of patient satisfaction. 

CUSTOMIZE was a Phase 3b trial which assessed the implementation of IM-LA CAB/RPV over 12 months in various American healthcare settings. After one year, 92% of participants preferred IM-LA injections to daily oral tablets and 97% planned to continue IM-LA treatment [82]. More long-term data in the real world are essential; implementation Phase 4 studies are ongoing (ILANA, CARISEL) [83].

In ALHIV populations, preliminary findings from MOCHA (More Options for Children and Adolescents) were presented at CROI 2022. MOCHA is a Phase I/II, open-label, non-comparative study of oral CAB, IM-LA CAB and IM-LA RPV in virologically suppressed ALHIV aged 12 to 18 years. The goal of the study was to confirm the dose, evaluate the safety, tolerability, acceptability, and pharmacokinetics of CAB when administrated as oral tablets, both IM-LA CAB and IM-LA RPV. Participants were assigned to receive either CAB or RPV additionally to their pre-study cART, starting with a 4 to 6-week oral lead-in phase followed by a 12-week injection phase in which participants received a total of either two or three injections, each administered four weeks apart. Interim results were based on 23 participants at week 16, showing that IM-LA 400 mg CAB and IM-LA 600 mg RPV were well tolerated and achieved concentrations comparable to those observed in adults when given individually [84]. The injection site reactions were either Grade 1 or Grade 2 and none led to the interruption of treatment. Adolescents and their parents/caregivers also took part in a qualitative evaluation of the tolerability, and the acceptability of the IM-LA ART injection perception was favorable; of the 21 adolescents who received three injections, 90.5% (19/21) indicated an interest in receiving IM-LA ARV even after the end of the study [85]. While these preliminary results are encouraging, more data are needed to confirm the safety, tolerability, acceptability, and pharmacokinetics of this treatment in adolescents. In March 2022, FDA approved the use of IM-LA CAB/RPV for the treatment of HIV-1 in virologically suppressed adolescents (≥18 years and ≥35 kg) with no known or suspected resistance to either CAB or RPV. Other issues surrounding CAB include the need for oral “lead-in” to ensure safety prior to injection of the IM formulation, and the theoretical risk of resistance resulting from the different half-lives of the two molecules. The long-term safety of this strategy remains to be established.

There is a promising perspective of directly using IM-LA CAB/RPV without “lead-in” (such as in adults [16,86]) in long-term-treated adolescents experiencing real and chronic difficulties in taking oral drugs. However, data are needed to prove the benefit of such strategy in ALHIV.

Interestingly, even among viremic adult patients in a real-world cohort, high rates of virologic suppression were observed with IM-LA CAB/RPV (with the same rate of failure as in registrational clinical trials of 1.5%) [87]. 

## 12. Peer-to-Peer Support

For physicians providing care to ALHIV, one of the keys to retaining them in care is the creation of an open, non-judgmental, flexible environment adapted to the ever-changing needs of adolescents. For these young adults, juggling school, work, family life and relationships are often overwhelming challenges, and it is difficult to attend clinical appointments. Clinics should be flexible in case of missed appointments or late arrival. Alternative strategies are needed to reach them, including the use of mobile technologies, like text messaging and smartphone applications with reminders. While in pediatric care, every effort is made to rescue them if they miss appointments; once in adult care, there is often limited follow-up and tracking after missed appointments. Some strategies to improve treatment effectiveness and adherence in ALHIV are summarized in Table 3. 

## 13. Transition to Adult Care

The transition of ALHIV from pediatric to adult care has been recognized as a major challenge, with numerous national and center-specific guidelines developed to attempt to address current gaps in care [88,89]. Nonetheless, many questions remain, including the optimal age for transition to adult care, the type of clinic to transfer to (adolescent clinics vs adult care), and the types of services to be included. The optimal age at transfer is potentially the one variable that can most easily be adapted to the needs of the individual patient. The main challenges after transfer to adult care are keeping young adults engaged in care, with continued virological suppression [90].

Age of transfer varies from one country to another. In Belgium, for example, there is no formal age for transfer. The decision is preferentially made when the adolescent is clinically stable, on a stable regimen with good adherence, virologically suppressed, and, most importantly, feels “ready” for transition. Prior to transfer, the young adult must be able to come by themself to the appointments, to be familiar with and to know the names of the different treatments they are taking, and to be able to file their own paperwork including requests for social assistance. One center has a specific transition clinic, while in another center, the same HIV nurses and social workers continue to follow the patients after transfer to adult care, after a co-consultation with the pediatrician and the adult HIV-physician chosen by the adolescent. 

In Canada, the official age of transfer is 18 years; a study showed that the majority (92%) of adolescents felt that 18 was too young for the transfer to adult care, and provided suggestions for improving the overall transition process [91]. In Montreal, Quebec the age at transition has become more flexible based on feedback from youth who have already been transferred to adult care. Regular “adolescent” groups are held, in which the process of transition is discussed between transferred patients and those who are about to transfer. Patients who fail to remain engaged in adult care are contacted by the pediatric clinic team and offered alternative choices. In our experience, it is very important to work with the young patient, obtain their feedback and focus on communication early in the process. The approach must be individualized, and while this may be feasible in rich-resource settings where numbers of ALHIV are low, this approach is more challenging in the most resource-limited countries [90]. A number of centers in the United States and Canada have also now extended the age of transition to 20–25 or beyond, with pediatric outpatient clinics continuing to follow youth on a case-by-case basis, despite official administrative directives that require transfer at the age of 18 [27]. In some countries, mostly in low- and middle-income countries, transition age can be much lower [92]. 

In all settings, it is a priority to promote adolescent-friendly services that combine both peer support (such as teen groups with common activities such as sport and artistic projects) and collaboration between health care providers to allow successful transitions into adulthood.

## 14. Impact of COVID 19 Pandemic

While HIV prevention, testing and antiretroviral treatment are essential measures to end the HIV epidemic, all of these were negatively affected during the COVID-19 pandemic due to the disrupted health care access [93]. Indeed, there was a significant decrease in HIV testing and diagnosis in 2020 in many countries [94,95,96,97], with a subsequent decrease cART initiation. For example, in the United States, HIV diagnoses as reported to the CDC decreased by 17% in 2020 compared with those reported in 2019 [96]. In a recent published study, changes in HIV testing and diagnoses among persons aged 1–14 years were assessed in the 22 President’s Emergency Plan for AIDS Relief (PEPFAR)-supported countries. HIV testing and case identification decreased by 40.1% and 29.4%, respectively [98]. One study also showed that the screening of sexually transmitted infections among adolescents was also impacted [95]. While historically, the majority of ALHIV acquired their infection through perinatal infection, new horizontal infections among adolescents are increasing and it represents a new challenge for prevention in the HIV epidemic. It is estimated that in 2020, 410,000 young people aged 10 to 24 were newly infected with HIV, accounting for nearly 30% of all new cases [99,100].

While published data remain limited, it can be inferred that the lack of testing and case identification may have significantly increased the risk of horizontal transmission in this group. Furthermore, on a global scale, the COVID-19 pandemic has also led to an increase in the overall prevalence of mental illness among children and adolescents [101,102]. 

## 15. Summary

While eliminating mother-to-child transmission of HIV is a realistic target of efforts to curb the HIV epidemic, any global child health gains made in reducing the number of vertically infected children will be offset by the challenges faced by the growing population of ALHIV. To allow adolescents the reaching of adulthood as healthy as possible, every effort should be made to optimize adherence and minimize side effects of ART in early childhood, while later in life, priorities include the development of adolescent-friendly clinics integrating group support, reproductive health and HIV prevention services, and organization of a safe transition to adult care.

## Figures and Tables

**Table 1:** Comparison of the different guidelines concerning initial regimen in ALHIV.

Guideline	PENTA/EACS	WHO Guidelines	NIH Guidelines
**Initial regimen**	ABC + 3TC + BIC or DTG	TDF + 3TC (or FTC) + DTG	TAF + FTC + DTG or BIC
**Alternative regimen**	TAF + 3TC + BIC or DTG	TDF + 3TC + EFV	ABC + 3TC + DTG or RAL

ABC, abacavir; BIC, bictegravir; DTG, dolutegravir; EFV, efavirenz; FTC, emtricitabine; RAL, raltegravir; TAF, tenofovir alafenamide; TDF, tenofovoir disoproxil fumarate; 3TC, lamivudine.

**Table 2:** Interactions of hormonal contraceptives with antiretroviral drugs (adapted from [50]).

Antiretroviral Class	Combined Hormonal Methods and Progestin-Only Pills	Levonorgestrel IUD	Progestin Implants	Progestin Injectables (DMPA)	Emergency Contraception
**Pis**	Interactions with most ritonavir-boosted PIs may decrease hormone contraceptive levels and contraceptive efficacy; alternative or additional contraceptive methods are recommended	No known interactions	Potential interaction, very limited clinical data	No known interactions	Possible interaction of ulipristal with elvitegravir/cobicistat; no clinical data
**NRTIs**	No known interactions	No known interactions	No known interactions	No known interactions	No known interactions
**NNRTIs**	Interactions with efavirenz may decrease hormonal contraceptive levels and contraceptive efficacy; alternative or additional contraceptive methods are recommended	No known interactions	Potential interaction with efavirenz may limit its contraceptive efficacy; more data are needed	No known interactions	Interactions of levonorgestrel emergency contraception with efavirenz may limit efficacy; possible interactions of ulipristal with NNRTIs; no clinical data
**Integrase inhibitors**	Possible interaction with elvitegravir/cobicistat	No known interactions	No known interactions	No known interactions	Possible interaction of ulipristal with elvitegravir/cobicistat
**Entry/fusion inhibitors**	No known interactions	No known interactions	No known interactions	No known interactions	No known interactions

**Table 3:** Approaches to optimize care and improve adherence in ALHIV.

Approach	Description
**Comprehensive Support**	Provide comprehensive support services tailored to the unique needs of adolescents living with HIV. Create a dedicated youth HIV clinic.
**Adherence Counseling**	Offer individualized counseling sessions to address adherence challenges and provide strategies for medication adherence.
**Peer Support**	Create support groups or peer networks for adolescents to connect, share experiences, and provide mutual encouragement.
**Youth-Friendly Services**	Design healthcare facilities and services that are teen-friendly, non-judgmental, and accessible for adolescents, with youth-friendly hours (e.g., evening hours).
**Mental Health Support**	Offer mental health services to address psychological and emotional well-being, including counseling and therapy.
**Medication Reminders**	Implement strategies such as mobile apps, SMS alerts, or pillboxes to help adolescents remember to take medications.
**Treatment Simplification**	Novel cART delivery strategies (e.g., long-acting oral or injectable cART), once daily/fixed-dose combinations, regimens to minimize side effects
**Family Involvement**	Involve families/caregivers in the treatment process, providing education and support to ensure adherence at home.
**Adherence Tools and Resources**	Provide educational materials, visual aids, and accessible resources to empower adolescents in managing their own care.
**Care Transition Planning**	Support a smooth transition from pediatric to adult healthcare services, ensuring continuity of care during the transition.
**Continuous Education**	Offer ongoing education and information on HIV, treatment advancements, and sexual health to empower informed decision-making.
**Sexual and Reproductive Health Education**	Provide comprehensive sexuality education, including information on safe sex, contraception, preventing transmission and U=U education.
